# CO_2_ embodied in trade: trends and fossil fuel drivers

**DOI:** 10.1007/s11356-020-12178-w

**Published:** 2021-01-29

**Authors:** Sylvain Weber, Reyer Gerlagh, Nicole A. Mathys, Daniel Moran

**Affiliations:** 1 University of Neuchâtel, Neuchâtel, Switzerland; 2 Tilburg University, Tilburg, Netherlands; 3 Federal Office for Spatial Development, Bern, Switzerland; 4 Norwegian University of Science and Technology, Trondheim, Norway

**Keywords:** International trade, Embodied emissions, Carbon leakage, Multi-region input-output analysis, Fossil fuels, Kyoto Protocol, F18, Q43, Q54, C67

## Abstract

The amount of CO_2_ embodied in trade has substantially increased over the last decades. We contribute to understanding the reasons for this evolution by studying the trends and some drivers of the carbon intensity of trade over the period 1995–2009 in 41 countries and 35 sectors. Our empirical analysis relies on the World Input-Output Database (WIOD) to compute embodied carbon emissions. Our main findings are the following. First, average emission intensity of traded goods is higher than average emission intensity of final demand. Second, relatively “dirty” countries tend to specialize in emission-intensive sectors. Third, the share of goods produced in emission-intensive countries is rising. Finally, we find that coal abundance (measured as fuel rent and controlling for reverse causality) leads both to a specialization in “dirty” sectors and to an increase in emissions per output when controlling for sector structure, which amounts to a fossil fuel endowment effect. These findings suggest trade liberalization may increase global emissions and therefore highlight the importance of considering trade when designing CO_2_ reduction strategies.

## Introduction

Carbon embodied in trade has increased dramatically over the last decades (see, e.g., IPCC 2014, chapter 5.4, Duarte et al. 2018, Li et al. 2020a, Yamano and Guilhoto 2020). Understanding the role of trade is therefore crucial to design effective international climate policies and avoid distortions in firms’ and countries’ incentives (Jakob and Marschinski 2013; Kander et al. 2015; Anouliès 2016; De Melo and Mathys 2010). This paper provides an empirical investigation of the trends and drivers of carbon intensity using a detailed input-output dataset of 15 years and more than 40 countries, thereby contributing to explaining this issue.

After the introduction of the Kyoto Protocol, it was suspected that carbon emissions could “leak,” in the sense that production of carbon-intensive goods could be relocated from Annex B countries (those with commitments in the Kyoto Protocol) to non-Annex B countries, and those goods could then be imported back to Annex B countries. If not coordinated, unilateral policies targeting emission reduction could then appear as effective at the country level but in fact be undermined or even counterproductive at the global level. In response to these concerns, consumption-based accounting (also called carbon footprint) stipulates that the final consumer of a good, rather than the producer, should be held accountable for emissions. Li et al. (2020b) make a similar case: large exporting countries such as China should strive for efficient production, whereas large importing countries such as the USA should implement policies to incentivize consumption of cleaner products. Implementing such a principle is challenging since it requires the representatives of final consumers to understand the mechanisms involved and have instruments to influence emissions up in the production chain, even if these emissions occur abroad.

**Fig. 1:**
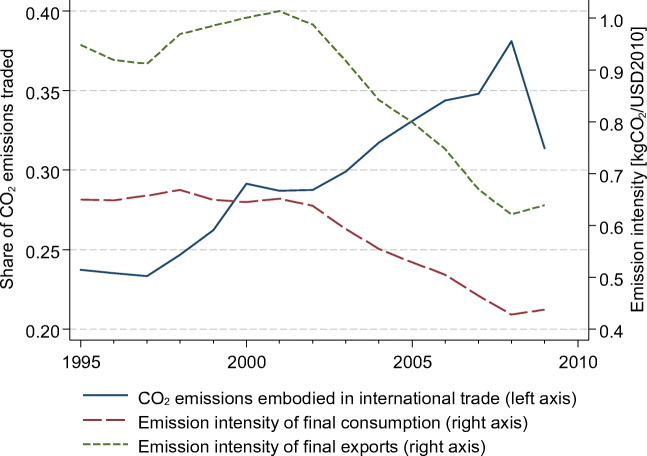
Evolution of the share of CO_2_ emissions embodied in international trade and of the emission intensity of final consumption and exports. Source: World input-output database (WIOD, Dietzenbacher et al. 2013b), own calculations

As shown in Fig. 1, carbon emissions embodied in trade constitute a substantial share of global emissions^1^. Over the 15-year observation window, this share has moreover risen from about one-quarter of global emissions to approximately one-third. This evolution mirrors the growth in the traded portion of global GDP over the same period. The declines observed during 1995–1997, 2000–2002, and (especially) 2008–2009 indicate that carbon flows are sensitive to global economic downturns (see Li et al. 2020a), but the long-run upward trend is expected to continue. Figure 1 additionally displays the development of emission intensities over time, for worldwide consumption and worldwide exports, respectively. We observe that emission intensities remained relatively stable between 1995 and 2002, and then rapidly declined. As also shown by Zhao and Liu (2020), traded goods tend to have substantially higher emission intensities, relative to the average final consumption, implying that the sheltered sectors have lower emission intensities. Service sectors with low emission intensities decrease the overall emission intensity of consumption, while their influence is minor for trade. It is therefore important to control for sector structure when investigating CO_2_ embodied in trade.

This paper belongs to the literature that studies the evolution of carbon emissions embodied in trade and their determinants. To be able to draw well-founded policy conclusions, we need a better understanding of emission intensities of production, consumption, and trade flows. To shed light on this issue, the novel approach we propose is to start by decomposing net CO_2_ exports into *trade deficits*, *sectoral structure* of the exporting country, and *average emission intensity* of the country. These three effects are equivalent to the scale, composition, and technique effects in the trade and environment literature (Grossman and Krueger 1993). Because we decompose net-embodied CO_2_ emissions in trade flows instead of total emissions, we use slightly different names for the components.

The relative importance of the three components and their relationships are interesting per se. For instance, if the latter two components are correlated, in the sense that emission-intensive countries tend to specialize in dirty sectors, increased trade would, everything else held equal, lead to increased emissions at the worldwide level. Yet, we go one step further and investigate determinants of sectoral structure and emission intensities. Following the literature (Aichele and Felbermayr 2012, 2015; Gerlagh et al. 2015; Grether et al. 2014; Michielsen 2013; Steckel et al. 2015), we focus on fossil fuel reserves and climate policies such as the Kyoto Protocol as potential drivers. The findings of our study therefore provide insights on the impacts of fossil fuel market developments and carbon policies on the evolution of emissions at the global level.

The remainder of the paper is structured as follows. The “Literature review” section gives an overview of the literature. The “Data and methodology” section describes the data used and the methodology applied to compute embodied carbon emissions. The “Results” section presents and discusses the results. The “Conclusions and policy implications” section formulates policy implications and concludes.

## Literature review

Our analysis builds on and combines several strands of the literature. First, it connects to the literature concerned with decomposing trade’s impact on emissions, and in particular to Grossman and Krueger’s (1993) influential contribution which decomposes the effect of trade on domestic emissions into three factors. The *scale* effect captures the mechanism whereby trade leads to increased economic activity and hence to increased emissions. The *composition* effect refers to a country’s sectoral specialization and implies that trade liberalization increases (decreases) domestic emissions when a country specializes in “dirty” (“clean”) sectors. The *technique* effect captures the mechanism whereby trade leads to more efficient production technologies, and thus to lower emissions. Using the above decomposition, Antweiler et al. (2001) conclude that increased trade tends to reduce SO_2_ concentrations. A number of further papers assess the link between trade and the environment. For instance, Cole (2006), Frankel and Rose (2005), and Managi et al. (2009) look at energy and trade, and also address endogeneity issues of trade and income. Some recent papers (e.g., Cole et al. 2014) use firm-level data but are limited to one or few countries.

In light of the growing importance of climate change, many studies have investigated the carbon content of global trade (Atkinson et al. 2011; Chen and Chen 2011; Davis and Caldeira 2010; Davis et al. 2011; Hertwich and Peters 2009; Peters and Hertwich 2008; Peters et al. 2011; Wiebe et al. 2012; Jiborn et al. 2018; Li et al. 2020a). These papers typically provide descriptive discussions (cf. Peters et al. 2011) without necessarily analyzing structural causes for the observed pattern of the carbon content of trade. A related strand of literature based on the Heckscher-Ohlin-Vanek model analyzes the factor content of trade and its determinants. It complements classic production factors (i.e., labor and capital) with environmental factors (e.g., Grether et al. 2012). We connect to this literature by considering fossil fuel endowments as explanatory variables for carbon embodied in trade.

More recently, theoretical extensions (Johnson and Noguera 2012; Trefler and Zhu 2010) and improvements in world input-output data became available. In particular, Grether and Mathys (2013) extend Antweiler’s (1996) work on the pollution terms of trade for SO_2_ with new and more detailed data. They find that large, poor, and emerging countries (i.e., Indonesia, China, Chile) exhibit high emission intensities for exports relative to imports, while large and rich countries (i.e., USA, Germany, Japan) are characterized by lower export emission intensities compared to their import emission intensities. Kanemoto et al. (2014) use the Eora input-output database to investigate the evolution of international flows of embodied CO_2_ and other greenhouse gases over the period 1970–2011. They conclude that global air pollution emissions have remained flat despite successful regulation in major emitters. In developed countries, air pollution footprints have increased, since reduced domestic emissions are more than offset by increased pollution embodied in imports.

Xu and Dietzenbacher (2014) exploit the World Input-Output Database (WIOD, Dietzenbacher et al. 2013b) and provide a dynamic structural decomposition analysis distinguishing emission intensities, trade structure of intermediate products, production technology, trade structure of final products, and total final demand. For many developed countries, the growth of emissions embodied in imports is found to be much higher than the growth of emissions embodied in exports, mainly because of changes in the structure of trade, both in intermediate and final products. They also observe that emerging economies like the BRIC countries have increased their share in global production and trade at the expense of developed countries, which tends to increase global average emission intensity. Su and Thomson (2016) also use WIOD to investigate the drivers of China’s changing carbon intensity of exports between 2006 and 2012, finding that exports become cleaner (i.e., their carbon intensity declines) but grow in total volume during that period. We use the same database and extend the analysis with an econometric approach allowing to uncover systematic relationships between economic growth and CO_2_ flows. Recently, Duarte et al. (2018) analyze the carbon embodied in bilateral trade flows among 39 countries from 1995 to 2009 based on WIOD. They find that countries such as the USA and Russia are displacing their pollution by importing growing volumes of CO_2_ from countries such as China, India, and Indonesia. Population and the level of development are two of the main contributors to CO_2_ displacement. Most recently, using network tools and WIOD, Li et al. (2020a) find increasing network density indicating widely expanding carbon leakages among economies. Also based on WIOD, Zhao and Liu (2020) analyze the factors influencing carbon emission intensities of different trade patterns. They find that emission intensity embodied in domestic trade is lower than that of international trade and that population, GDP per capita, energy intensity, and trade are significant determinants. They suggest that international organizations should consider the transfer of carbon emissions through international trade and reasonably allocate reduction responsibilities between consumers and producers. Analyzing consumption-based carbon emissions in sub-Saharan Africa, Adams and Opoku (2020) come to similar policy conclusions.

Aichele and Felbermayr (2012, 2013) evaluate the effect of the Kyoto Protocol on carbon embodied in trade. Controlling for the endogeneity of Kyoto Protocol commitments, they find that embodied carbon imports from non-committed to committed countries have increased by around 8% and emission intensity of these imports have increased by about 3%. In the same vein but applied to the energy content of trade and looking at energy endowments as determinants of comparative advantages, Gerlagh et al. (2015) find for a high-income country sample that a one standard deviation increase in energy abundance raises energy embodied in trade by about 20%. The authors also find that energy-abundant countries have 7–10% higher employment and 13–17% higher net exports in energy-intensive sectors vis-à-vis otherwise comparable countries. Sato and Dechezleprêtre (2015) study the effect of energy prices on trade for a panel of 42 countries. Estimating a gravity equation for the carbon content of trade, they find statistically significant but very small effects of energy prices on trade flows. Douglas and Nishioka (2012) test theoretical predictions from the Heckscher-Ohlin-Vanek and Trefler and Zhu (2010) framework. They find no evidence that developing countries specialize in emission-intensive sectors. Instead, evidence suggests that emission intensities differ systematically across countries because of differences in production techniques. Results confirm that international differences in emission intensity are substantial but also suggest that the latter do not play a significant role in determining trade patterns. We build on this literature, using a comprehensive worldwide input-output dataset. Our main contribution is to provide an empirical strategy that allows estimating the three components of net embodied carbon emissions in trade: trade deficits, sectoral structure, and average emission intensities. Furthermore, our approach highlights the underlying importance of fossil fuel endowments.

## Data and methodology

### Data

Our empirical analysis makes use of data on production, trade, consumption, sectoral CO_2_ emissions, and carbon footprints from the World Input-Output Database (WIOD) (Dietzenbacher et al. 2013b; Timmer 2012), which pertains to a new generation of global trade databases for tracing flows of carbon embodied in trade along the whole value chain. WIOD was chosen over the EXIOBASE (Tukker et al. 2013), Eora (Lenzen et al. 2012; Lenzen et al. 2013; Kan et al. 2020), and GTAP (Andrew and Peters 2013; Narayanan et al. 2012) because of its homogenous sector classification and its sectoral, spatial, and temporal detail and coverage. The time span 1995–2009 is of interest since developing countries lowered significantly their average tariffs. For a discussion of the relative strengths and weaknesses of these databases, see Dietzenbacher et al. (2013a), Owen et al. (2014), and Tukker and Dietzenbacher (2013). WIOD covers 41 countries (listed in Appendix 3,3) each containing 35 sectors (listed in Appendix Table 4) over the period 1995–2009. Deducting a few missing observations, this leads to a dataset of roughly 20,000 observations when the sectoral dimension is used and roughly 600 observations when sectors are aggregated at the country level. Our research questions focus on the country (and not the country-pair) perspective. Only Eqs. (8), (11), and (12) in the “Determinants of emission intensities” section, and the corresponding results in the Appendix are performed at the country-pair level.

Income per capita is taken directly from WIOD, and further variables from other sources are used to complement the database. Income, population, and natural resource rents are taken from the WIOD database and the World Development Indicators (World Bank). A dummy variable is also used to indicate whether or not a country has ratified the Kyoto Protocol in a given year. As an alternative to the latter indicator, a CO_2_ stringency index is borrowed from Sauter (2014) and constructed by counting supra-national, national, and sub-national laws, which explicitly refer to the goal of reducing CO_2_ emissions.

### Empirical methodology

Our empirical methodology derives from a standard input-output analysis (see, e.g., Miller and Blair 2009 for an extensive presentation). In this framework, CO_2_ emissions from sector *s* of country *i* can be expressed as territorial emissions *T* (also known as production-based) or consumption-based emissions *C* as follows:^2^

1 $$ {T}_{is}={e}_{is}{x}_{is}={\varepsilon}_{is}{z}_{is} $$

2 $$ {C}_{is}={\varphi}_{is}{y}_{is} $$

where *e* represents emission intensity of output, i.e., the quantity of CO_2_ emitted per unit of output, *x* represents output, *ε* represents emission intensity of value added, *z* represents value added, *φ* is emission intensity of final demand including embodied carbon emissions (both from national and international intermediate goods), and *y* is final demand. Note that ${\sum}_{is}{T}_{is}={\sum}_{is}{C}_{is}$ by definition.

**Fig. 2:**
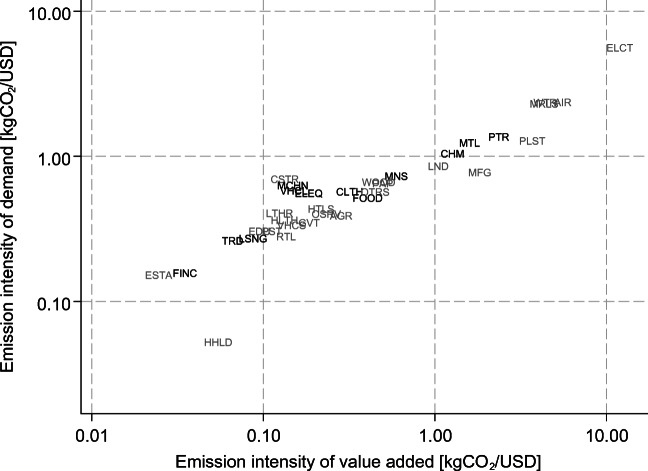
Emission intensities by sector, in 2009. Note: See Table 4 in the Appendix for full sector names. Axes use logarithmic scaling

Fig. 2 plots emission intensities of a typical sector in a typical country in 2009, the most recent year available in the dataset. These values were obtained by regressing emission intensities on time fixed effects, country-time fixed effects normalized on average to zero in each year, and sector-time fixed effects normalized on average to zero in each year. Dark labels indicate trade-intensive sectors (i.e., sectors with exports above average), while light labels indicate sheltered sectors (i.e., sectors with exports below average). Emission intensities of value added (*ε*) are shown on the horizontal axis, while emission intensities of demand (*φ*) are presented on the vertical axis (both axes in logarithmic scale). We observe that a few sectors are much more emission-intensive than all others. In particular, “Electricity, Gas and Water Supply” (ELCT), “Air Transport” (AIR), “Other non-metallic minerals” (MRLS), and “Water Transport” (WTR)^3^ are classified as the most emission-intensive sectors, both in terms of value added and consumption. No clear-cut picture emerges at this stage concerning the degree of trade exposure and emission intensity. These results do not seem to be driven by the 2008–2009 economic crisis as they also hold for the other years of the observation period.

At first glance, this finding might seem at odd with Fig. 1, which shows that exports are more emission-intensive than final demand. This apparent contradiction is explained as follows: though the most emission-intensive sectors are sheltered, they are small compared to the next group of emission-intensive traded sectors. Specifically, the top 4 sectors in terms of emission intensities (ELCT, AIR, MRLS, WTR) make up only 3.5% of total worldwide final demand in 2009, and thereby contribute a limited amount to the average emission intensity of final demand. Among the trade-intensive sectors, “Coke, Refined Petroleum and Nuclear Fuel” (PTR), “Basic Metals and Fabricated Metal” (MTL), and “Chemicals and Chemical Products” (CHM) are the most emission-intensive sectors, making up 19.3% of total worldwide exports. Hence, these sectors are relatively exposed to trade, large and relatively emission-intensive.

At the world level, *T* = *C* by definition, but the two measures differ when for individual countries and individual sectors. For each country *i*, net CO_2_ exports (*NCO*2*XP*) can then be expressed as:^4^

3 $$ {\displaystyle \begin{array}{c} NCO2{XP}_i={T}_i-{C}_i={\boldsymbol{e}}_{\boldsymbol{i}}^{\prime }{\boldsymbol{x}}_{\boldsymbol{i}}-{\boldsymbol{\varphi}}_{\boldsymbol{i}}^{\prime }{\boldsymbol{y}}_{\boldsymbol{i}}={\boldsymbol{\varphi}}_{\boldsymbol{i}}^{\prime}\left(\boldsymbol{I}-{\boldsymbol{A}}_{\boldsymbol{i}}\right){\boldsymbol{x}}_{\boldsymbol{i}}-{\boldsymbol{\varphi}}_{\boldsymbol{i}}^{\prime }{\boldsymbol{y}}_{\boldsymbol{i}}\\ {}={\boldsymbol{\varphi}}_{\boldsymbol{i}}^{\prime}\left[\left(\boldsymbol{I}-{\boldsymbol{A}}_{\boldsymbol{i}}\right){\boldsymbol{x}}_{\boldsymbol{i}}-{\boldsymbol{y}}_{\boldsymbol{i}}\right]={\boldsymbol{\varphi}}_{\boldsymbol{i}}^{\prime}\left({\boldsymbol{XP}}_{\boldsymbol{i}}-{\boldsymbol{MP}}_{\boldsymbol{i}}\right)\end{array}} $$

where ***I*** is an identity matrix, ***A***_***i***_ is the input-output coefficients matrix, i.e., a matrix where each column indicates the inputs from all sectors needed to produce one unit of output in a given sector, ***XP***_***i***_ are exports from country *i* and ***MP***_***i***_ are imports including imports of intermediate goods to country *i*. We decompose net CO_2_ exports (adapting the decomposition by Grossman and Krueger 1993, to the net emission content of trade) into the following three components: trade balance (reflecting the scale effect), sector specialization (reflecting the composition effect), and country-specific emission intensities (reflecting the technique effect):

4 $$ NCO2{XP}_i=\overline{\varphi}{\boldsymbol{u}}^{\prime}\left({\boldsymbol{XP}}_{\boldsymbol{i}}-{\boldsymbol{MP}}_{\boldsymbol{i}}\right)+\left({\overline{\boldsymbol{\varphi}}}_{\boldsymbol{s}}^{\prime }-\overline{\varphi}{\boldsymbol{u}}^{\prime}\right)\left({\boldsymbol{XP}}_{\boldsymbol{i}}-{\boldsymbol{MP}}_{\boldsymbol{i}}\right)+\left({\boldsymbol{\varphi}}_{\boldsymbol{i}}^{\prime }-{\overline{\boldsymbol{\varphi}}}_{\boldsymbol{s}}^{\prime}\right)\left({\boldsymbol{XP}}_{\boldsymbol{i}}-{\boldsymbol{MP}}_{\boldsymbol{i}}\right) $$

where ***φ***_***i***_ is the vector of sectoral emission intensities of demand in country *i* (this is also known as the Leontief multiplier or embodied emissions intensity), ${\overline{\boldsymbol{\varphi}}}_{\boldsymbol{s}}$ is the vector of world average emission intensities per sector, $\overline{\varphi}$ is the average emission intensity over all sectors and all countries (i.e., a scalar), and ***u*** is a vector of ones.

The first term on the right-hand side of (4) represents the net CO_2_ trade related to the economic trade balance. This term uses a worldwide average emission intensity of goods. Countries exporting much more than they import, such as China, tend to have a positive first term.

The second term represents the net CO_2_ trade position related to the sector structure of exports and imports. The term is positive if a country exports in sectors that tend to be emission-intensive and/or it imports in sectors that tend to have low associated emissions. The second term is closely related to the pollution haven debate (Duarte et al. 2018).

The third term represents the net CO_2_ trade related to differences in the emission intensities between the (exporting) country *i* and its importing partners. The term is positive if domestic emission intensities exceed the sector world average and/or if the foreign emission intensities from which the country imports are below the sector world average. This term is thus expected to be positive for countries with “inefficient” domestic production, and for countries whose trade partners are emission-efficient. This term measures overall production efficiency of a country *relative* to its trading partners. A country such as the USA may be emission-intensive compared to the EU, but if it trades more intensely with China, then its relative performance to China matters more for its net trade in CO_2_ position.

We consider the decomposition in (4) over time in order to identify how the contributions of the three factors evolve. Moreover, looking at the correlations between the different components and their evolution over time indicates whether trade tends to increase or decrease worldwide emissions. For example, a positive correlation between sector specialization and emission intensities (second and third terms) would imply that CO_2_-intensive countries specialize in CO_2_-intensive sectors, and more trade is then accompanied by more emissions. Also, if emission-intensive countries tend to exhibit a trade surplus, worldwide emissions would increase with trade, everything else equal. The results of this decomposition are reported and discussed in the “Decomposing CO_2_ embodied in trade” section below.

### Determinants of emission intensities

**Fig. 3:**
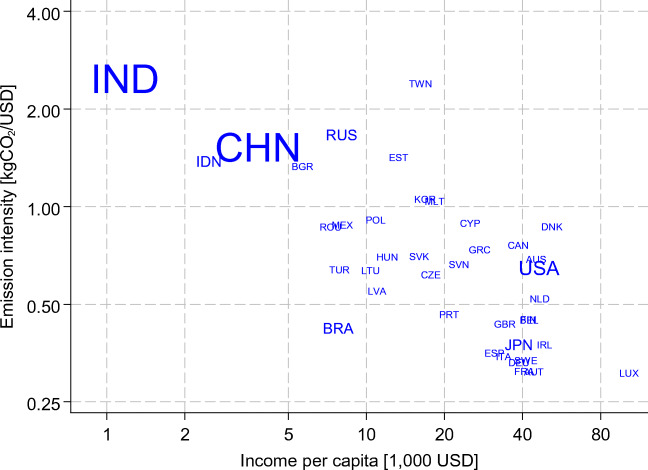
Emission intensity of value added versus income, 2009. Note: Size of marker proportional to population

Fig. 3 displays the relation between income and emission intensity of value added. It shows that production in high-income countries tends to be more emission-efficient compared to that in low-income countries. However, for a given income level, there is wide variability in the emission intensity of production.

**Fig. 4:**
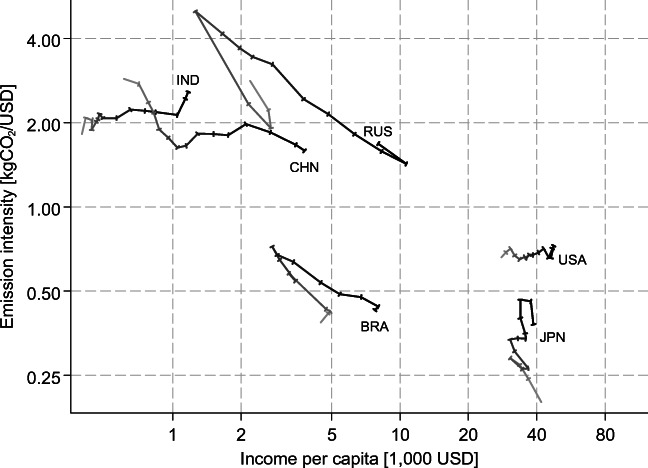
Emission intensity of value added versus income for some selected (largest) countries, 1995–2009. Note: Connectors indicate the annual moves from 1995 (light) to 2009 (dark)

Fig. 4 displays the evolution of emission intensities for some large countries. While emission intensities increase and then decrease over the years for Russia and Brazil, they increase (almost) continuously for India and Japan, and decrease (almost) continuously for China.^5^ The USA does not show any significant change in emission intensities. While income is clearly negatively correlated with the level of emission intensities across countries (Fig. 3), the evolution of this relationship within countries over time is much less obvious (Fig. 4).

In order to investigate if and how income, fuel markets, climate policies, and trade opportunities drive changes in emission intensities and in trade patterns, we use the following specifications:

5 $$ \mathrm{EIVA}:\ln \left({\varepsilon}_{ist}\right)={\beta}^{VA}{Z}_{it}+{\gamma}_i+{\delta}_{st}+{\mu}_{ist} $$

6 $$ \mathrm{EID}:\ln \left({\varphi}_{ist}\right)={\beta}^D{Z}_{it}+{\gamma}_i+{\delta}_{st}+{\mu}_{ist} $$

where *ε*_*ist*_ is emission intensity of value added (EIVA) in sector *s* of country *i* at time *t*, *φ*_*ist*_ is emission intensity of demand inclusive of embodied emissions (EID), *Z*_*it*_ includes country variables such as income, fossil fuel income shares, and policies. The effect of these variables is identified through different trends between countries, as time fixed effects are absorbed by the sector-time fixed effects *δ*_*st*_, and time-invariant country characteristics are absorbed through country fixed effects *γ*_*i*_, while *μ*_*ist*_ is the remaining idiosyncratic noise. Depending on the variables included in *Z*_*it*_, the estimated coefficients *β* can answer questions such as whether domestic fossil fuel abundance, Kyoto policies, and trade opportunities tend to increase or decrease emission intensities.

To gain further insights, we test alternative measures of emission intensity that are relevant in the context of trade:

7 $$ \ln \left(\frac{{\boldsymbol{\varphi}}_{\boldsymbol{it}}^{\prime }{\boldsymbol{XP}}_{\boldsymbol{it}}}{{\overline{\boldsymbol{\varphi}}}_{\boldsymbol{st}}^{\prime }{\boldsymbol{XP}}_{\boldsymbol{it}}}\right)={\beta}_1^X\ {Z}_{it}+{\gamma}_i+{\delta}_t+{\mu}_{it} $$

8 $$ \ln \left(\frac{{\boldsymbol{\varphi}}_{\boldsymbol{it}}^{\prime }{\boldsymbol{XP}}_{\boldsymbol{ijt}}}{{\overline{\boldsymbol{\varphi}}}_{\boldsymbol{st}}^{\prime }{\boldsymbol{XP}}_{\boldsymbol{ijt}}}\right)={\beta}_2^X\ {Z}_{it}+{\gamma}_i+{\delta}_{jt}+{\mu}_{ijt} $$

The left-hand side variable in (7) measures emissions of country *i* exports (computed as the product between the (transposed) vector of country-sector emission intensities with the corresponding export flows), relative to emissions for an average country (i.e., using the world average vector for emission intensity) with the same sector structure of exports (i.e., multiplying with country *i*’s export structure). The dependent variable in (8) is similar, but specified for each bilateral country-pair: ***XP***_***ijt***_ represents exports from country *i* to country *j* during year *t*. In this case, we control for country and partner-year fixed effects. These two dependent variables are closely related to the third term of (4) and these two equations will give insights in the factors explaining country-specific emission intensities.

We then investigate the sectoral structure of trade by estimating the following four equations:

9 $$ \ln \left(\frac{{\overline{\boldsymbol{\varphi}}}_{\boldsymbol{st}}^{\prime }{\boldsymbol{XP}}_{\boldsymbol{it}}}{{\overline{\varphi}}_t{\boldsymbol{u}}^{\prime }{\boldsymbol{XP}}_{\boldsymbol{it}}}\right)={\beta}_1\ {Z}_{it}+{\gamma}_i+{\delta}_t+{\mu}_{it} $$

10 $$ \ln \left(\frac{{\overline{\boldsymbol{\varphi}}}_{\boldsymbol{st}}^{\prime }{\boldsymbol{MP}}_{\boldsymbol{it}}}{{\overline{\varphi}}_t{\boldsymbol{u}}^{\prime }{\boldsymbol{MP}}_{\boldsymbol{it}}}\right)={\beta}_2\ {Z}_{it}+{\gamma}_i+{\delta}_t+{\mu}_{it} $$

11 $$ \ln \left(\frac{{\overline{\boldsymbol{\varphi}}}_{\boldsymbol{st}}^{\prime }{\boldsymbol{XP}}_{\boldsymbol{ijt}}}{{\overline{\varphi}}_t{\boldsymbol{u}}^{\prime }{\boldsymbol{XP}}_{\boldsymbol{ijt}}}\right)={\beta}_3\ {Z}_{it}+{\beta}_4{Z}_{jt}+{\gamma}_i+{\lambda}_j+{\delta}_t+{\mu}_{ijt} $$

12 $$ \ln \left(\frac{{\overline{\boldsymbol{\varphi}}}_{\boldsymbol{st}}^{\prime }{\boldsymbol{XP}}_{\boldsymbol{ijt}}}{{\overline{\varphi}}_t{\boldsymbol{u}}^{\prime }{\boldsymbol{XP}}_{\boldsymbol{ijt}}}\right)-\ln \left(\frac{{\overline{\boldsymbol{\varphi}}}_{\boldsymbol{st}}^{\prime }{\boldsymbol{XP}}_{\boldsymbol{jit}}}{{\overline{\varphi}}_t{\boldsymbol{u}}^{\prime }{\boldsymbol{XP}}_{\boldsymbol{jit}}}\right)={\beta}_5\ \left({Z}_{it}-{Z}_{jt}\right)+\left({\gamma}_i-{\gamma}_j\right)+{\mu}_{ijt} $$

All dependent variables in these equations are measures of sector structure and are linked to the second term in (4). The dependent variable in (9) measures the sector bias of exports towards emission-intensive sectors, i.e., how the export structure of country *i* causes its emission intensity to differ from the average. In (10), we consider an equivalent variable for imports. In (11), the dependent variable measures the sector bias for all country-pairs of bilateral trade, considering each country-pair in both ways (*i* is both an exporter to *j* and an importer from *j*). Coefficient *λ*_*j*_ represents partner fixed effects. In (12), we subtract exports from country *j* to country *i* (imports in country *i* from country *j*) from exports from country *i* to country *j* to obtain a symmetric indicator equivalent to (11) for relative emission intensities in net exports (export-imports). We expect *β*_5_ to be about equal to *β*_3_ − *β*_4_. Note that the country-partner fixed effects in (12) are structured so that their number is equal to the number of countries, and not to the number of country-partner pairs.

### Controlling for unobserved endogeneity and weighting observations

The objective of our analysis is to investigate whether an increase in fossil fuel rents (e.g., coal) tends to increase or decrease the emission intensity of production (5), consumption (6), and exports (7)-(8), and whether it alters the sector structure of trade (9)-(12). However, reverse causality could also arise: an increased demand for emission-intensive sectors leads to higher fossil fuel prices, and thus to higher fossil fuel rents. Therefore, we implement a “shift-share” approach similar to that in Allcott and Keniston (2018) and Bartik (1991). For each country, we calculate the share of that country *i*, over the entire period, in worldwide fuel rents: ${s}_i^c$. In addition, for each year *t*, we calculate the global fuel rents as a share of world GDP: ${R}_t^g$. The interaction between the country’s share and the world fuel rents is used as an independent variable instead of the country’s fossil fuel rent:

13 $$ {R}_{it}={s}_i^c{R}_t^g $$

By construction and assuming that country *i*’s influence on total world resource rents is sufficiently small, this interaction cannot suffer from reverse causality: an increase in fossil fuel demand in one country in 1 year will have no effect on the interaction term for that country in that year. This seems a plausible assumption to the best of our knowledge.

We also use trade openness as an independent variable in our estimations. Similarly, to avoid endogeneity, we consider openness in our estimations through the interaction between a country’s average openness over the entire period and the world trade share in world GDP, for each year.

The approach outlined above, inspired by Allcott and Keniston (2018), uses the interactions between the country’s share and the world fuel rents directly in the equation of interest. This methodology relies on a single-equation methodology, and therefore avoids any cross-influences of the various instruments on the endogenous variables taking place in a standard two-stage approach.^6^

Depending on the dimensions of the dependent variable (country-sector-year-partner), we use the corresponding fixed effects in order to control for unobserved heterogeneity (see results’ tables for details). We do not include scaling variables such as population or GDP since our dependent variable reflects emission intensities and not emissions.

We conduct both weighted and unweighted regressions. Weighting is warranted if we expect observations concerning large trade flows to have better quality, in relative terms, compared to observations concerning small trade flows. Another way to interpret differences between weighted and unweighted estimations is that the former indicates marginal effects for the weighted average observation, while the latter applies to the unweighted average observation. The two outcomes will differ when large countries behave systematically differently compared to smaller ones.

## Results

### Decomposing CO_2_ embodied in trade

**Fig. 5:**
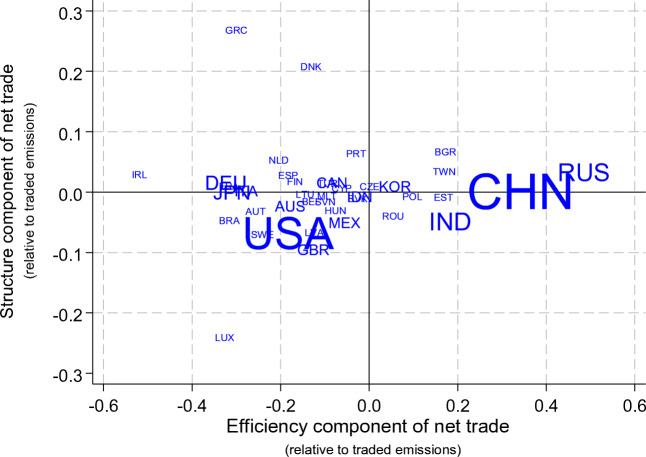
Contribution of domestic efficiency compared to trade partners and sector structure of trade, to net CO_2_ exports, 2009. Note: Size of marker proportional to territorial CO_2_ emissions. Note the different scales on the horizontal and vertical axis

Fig. 5 illustrates the decomposition of net CO_2_ exports presented in (Eq. 4) by plotting the sector structure effect (second term) against the efficiency effect (third term) for all countries in our sample. Two countries, the USA and China, have the largest net CO_2_ trade positions, as indicated by the size of their marker. However, when total trade is accounted for, China and Russia stand out as net CO_2_ exporters because of their emission-intensive production, whereas the size of US CO_2_ inflows is relatively moderate compared to the amount of its domestic emissions. These findings are consistent with Li et al. (2020b), who show that energy flows embodied in Sino-US bilateral trade are the largest in the world and these are heavily imbalanced, with energy embodied in trade flows from China to USA being much larger than in the opposite direction.

### Patterns in emission intensity and trade specialization

To investigate how income, fossil fuel abundance, trade opportunities, and Kyoto affect emission intensity of production and trade, we estimate the series of regressions presented in Eqs. (5) to (12). These relationships are ambiguous, so that a thorough empirical analysis is warranted. In particular, the effect of Kyoto is much debated in the literature. For instance, while Almer and Winkler (2012) find no effect of being committed to an emission target under the Kyoto Protocol, Aichele and Felbermayr (2013) obtain robust evidence that Kyoto commitment reduced CO_2_ emissions. In addition to the environmental policy variable Kyoto, we follow the literature and account for fossil fuel rents as a share of GDP and trade openness as additional independent variables.

Results are displayed in Table 1. The first row shows that there is a well-established substantial negative effect of income on emission intensity. The efficiency improvement, however, does not catch up with income since the elasticity is significantly smaller than one in absolute value. Thus, even though emission intensity decreases with income, overall emissions nevertheless increase robustly with income. Moreover, the second-order effect of income is small in size, which is in contradiction with the hypothesis of an environmental Kuznets curve.^7^

**Table 1:** Determinants of emission intensities of value added, output and exports, controlling for sector structure

Dependent variable	EIVA	EID	EI exports	EI exports
Equation number	(5)	(6)	(7)	(8)
ln(income)	− 0.801***	− 0.707***	− 0.652***	− 0.604***
	(0.035)	(0.019)	(0.020)	(0.007)
ln(income)^2^	− 0.013	− 0.020***	− 0.007	-
	(0.008)	(0.005)	(0.005)	
Rents coal	0.019*	0.014**	0.018***	0.020***
	(0.011)	(0.006)	(0.006)	(0.002)
Rents oil	0.019*	0.005	0.019***	0.025***
	(0.010)	(0.005)	(0.005)	(0.002)
Rents gas	− 0.019*	− 0.022***	− 0.016***	− 0.028***
	(0.011)	(0.006)	(0.006)	(0.003)
Trade	0.378**	0.238***	0.080	− 0.310***
	(0.158)	(0.085)	(0.072)	(0.029)
Kyoto	0.054***	0.082***	0.024*	-0.029***
	(0.019)	(0.010)	(0.013)	(0.007)
Country FE	YES	YES	YES	YES
Partner-year FE	NO	NO	NO	YES
Sector-year FE	YES	YES	NO	NO
Year FE	NO	NO	YES	NO
Weights	YES	YES	YES	YES
*N*	19,430	19,861	585	11,108
*R*^2^	0.880	0.899	0.987	0.981
*R*^2^ within	0.089	0.128	0.748	0.071

Note: Standard errors in parentheses. */**/***: significant at 10/5/1%

We find that coal abundance significantly increases emission intensity. A one percentage point increase in coal rents, as a share of GDP, increases relative emission intensity of value added and exports by about 2%. Evidence is similar for oil. Contrarily, for natural gas, we find a negative effect: gas-abundant countries tend to become less emission-intensive in years of high gas prices. These results reflect the relative carbon intensity of fuels, with gas being less carbon-intensive than oil and coal.

Concerning trade and climate policies, results vary across specifications. Also, weighted (Table 1) and unweighted estimates (Table 6 in the Appendix) lead to different results as long as trade partners are not controlled for (Eq. 8) controls for trade partners). In the latter specification, both trade openness and Kyoto commitment lead to a decrease of export emission intensities. As an alternative to the Kyoto variable, we also used a CO_2_ index as proposed by Sauter (2014) in a series of robustness checks (see Tables 10 and 11 in the Appendix). We have not controlled for the potential endogeneity of the Kyoto Protocol or CO_2_ index, given that identifying the causal impact of these measures is not our primary objective in this paper. Note although that Aichele and Felbermayr (2012) obtain very similar results for the Kyoto variable using both OLS and instrumental variable estimations.

Table 2 displays the results obtained for Eqs. (9) to (12) and allows to investigate the drivers for the sectoral composition of trade. High-income countries tend to specialize in emission-intensive sectors, as exports in these sectors are found to increase when we do not control for the trading partners (Eq. 9). When controlling for trading partners though (Eqs. 11a) and (12), high-income countries seem to specialize in emission-extensive sectors. These results are suggestive of the following pattern. High-income countries have comparative advantages in emission-extensive sectors but they also trade more with other high-income partners who demand imports from emission-intensive sectors 11b). The net effect of an income increase is an increase in the emission intensity of exports (9). Results obtained using unweighted estimations (Table 7 in the Appendix) confirm this pattern. When controlling for trade partners, high-income countries have lower emission intensities in both imports and exports, with a stronger effect for exports. Without this control, higher income countries have higher emission intensities in imports and exports.

**Table 2:** Effects on sector structure

Dependentvariable	Exports(separate)	Imports (separate)	Exports (joint)	Imports (joint)	Exports-imports
Equation number	(9)	(10)	(11a)	(11b)	(12)
ln(income)	0.050***	0.013	− 0.033***	0.045***	− 0.070***
	(0.011)	(0.009)	(0.009)	(0.009)	(0.007)
ln(income)^2^	0.001	− 0.006***	-	-	-
	(0.003)	(0.002)			
Rents coal	− 0.006*	0.002	0.012***	− 0.006***	0.013***
	(0.003)	(0.003)	(0.002)	(0.002)	(0.002)
Rents oil	0.002	− 0.002	− 0.004*	0.011***	− 0.013***
	(0.003)	(0.002)	(0.002)	(0.002)	(0.002)
Rents gas	0.007**	0.002	− 0.010***	− 0.009***	− 0.002
	(0.003)	(0.003)	(0.003)	(0.003)	(0.002)
Trade	0.110***	0.090***	0.142***	− 0.177***	0.234***
	(0.039)	(0.031)	(0.031)	(0.031)	(0.026)
Kyoto	− 0.002	0.010**	0.029***	− 0.016***	0.000
	(0.007)	(0.005)	(0.005)	(0.005)	(0.004)
Country FE	YES	YES	YES	YES	JOINT
Partner FE	NO	NO	YES	YES	JOINT
Year FE	YES	YES	YES	YES	NO
Weights	YES	YES	YES	YES	YES
*N*	585	585	22,709	22,709	11,017
*R*^2^	0.932	0.904	0.938	0.938	0.458
*R*^2^ within	0.145	0.108	0.059	0.059	0.066

Note: Standard errors in parentheses. */**/***: significant at 10/5/1%. All regressions are weighted by trade flows

The estimates also tend to show that coal abundance leads to specialization in dirty sectors, while oil and gas abundance lead to specialization in relatively clean sectors. Increased trade leads to an unambiguous increase in the share of emission-intensive sectors. Not only are the traded goods more emission-intensive, compared to the average good, but increased trade amplifies the difference. This result is confirmed in unweighted estimations.

Kyoto ratification is positively correlated with an increase in imports of emission-intensive sectors (10), but not when controlling for the trading partner (11b). This finding suggests a shift in trading partners, following Kyoto ratification, as a potential consequence of reducing domestic emissions. The effect on exports, controlling for trading partners, is different for weighted versus unweighted estimates. There seems to be a structural difference between large and small countries.

## Conclusions and policy implications

Trade shall be considered when designing greenhouse gas mitigation policies. Indeed, global emissions would not decline if countries export their emissions outside of a regulatory zone, and it is not desirable that domestic abatement policies are undermined by carbon-intensive imports. Countries with relatively ambitious climate policies should therefore be aware of the potentially large amount of imported emissions and they should carefully consider the relevant drivers. On the other side, emerging economies are traditionally net exporters of embodied emissions. These countries face a trade-off between promoting growth and limiting their climate impact. Our findings are helpful to trade and climate policy design in both types of countries, and it appears crucial to have good understanding of the trends and drivers of CO_2_ embodied in trade.

Our findings show that trade-exposed sectors are more emission-intensive than sheltered sectors, and that intensifying trade tends to further increase the emission intensity of traded goods. One possible mechanism underlying this positive correlation is based on fossil fuels as production factors. In fact, we find that coal abundance leads both to a specialization in “dirty” sectors and to an increase of emissions per output when controlling for sector structure: a fossil fuel endowment effect.

Considering trade and paying due attention to fossil fuel markets, specifically coal, appears crucial when designing CO_2_ reduction strategies. Many of the most carbon-intensive countries are also developing economies. With economic growth, emission intensity tends to decline, but insufficiently to compensate the direct effect of income on emissions. The net effect of an income rise is thus to increase overall emissions. Although our analysis does not offer immediate solutions to disconnect income growth and increased trade from increased emissions, it offers some insights into the drivers, and as such, is helpful to pave the way for future effective measures.

Our results are also relevant for international organizations, such as the WTO, the OECD, and the UN, working on international cooperation in terms of climate and trade policies. Better understanding linkages among trade partners and the importance of fossil fuel drivers allows them to propose empirically sound and comprehensive policy measures. With the principle of “common but differentiated responsibilities” the Kyoto Protocol ignited a discussion about a “fair” allocation of greenhouse gas emission rights and the corresponding mitigation costs. Meetings of the Conference of the Parties (COPs) have largely discussed this interpretation. Our results highlight that carbon leakage effects must be present in these discussions.

## Data Availability

The datasets analyzed during the current study are available and described here: http://www.wiod.org/home Dietzenbacher E, Los B, Stehrer R, Timmer M and de Vries G 2013b “The construction of world input-output tables in the WIOD project” Economic Systems Research 25(1): 71-98 https://databank.worldbank.org/source/world-development-indicators Sauter C 2014 “How should we measure environmental policy stringency? A new approach” University of Neuchâtel, Institute of Economic Research, Working paper 14-01.

## Appendix 1. Definitions and abbreviations

**Table 3:** Country list (WIOD)

#	Country code	Country
1	AUS	Australia
2	AUT	Austria
3	BEL	Belgium
4	BRA	Brazil
5	BGR	Bulgaria
6	CAN	Canada
7	CHN	China
8	CYP	Cyprus
9	CZE	Czech Republic
10	DNK	Denmark
11	EST	Estonia
12	FIN	Finland
13	FRA	France
14	DEU	Germany
15	GRC	Greece
16	HUN	Hungary
17	IND	India
18	IDN	Indonesia
19	IRL	Ireland
20	ITA	Italy
21	JPN	Japan
22	LVA	Latvia
23	LTU	Lithuania
24	LUX	Luxembourg
25	MLT	Malta
26	MEX	Mexico
27	NLD	Netherlands
28	POL	Poland
29	PRT	Portugal
30	ROM	Romania
31	RUS	Russia
32	SVK	Slovakia
33	SVN	Slovenia
34	KOR	South Korea
35	ESP	Spain
36	ROW	Rest of World
37	SWE	Sweden
38	TWN	Taiwan
39	TUR	Turkey
40	GBR	UK
41	USA	USA

**Table 4:** Sector list (WIOD)

#	Sector code	Sector
1	AGR	Agriculture, hunting, forestry, and fishing
2	AIR	Air transport
3	MTL	Basic metals and fabricated metal
4	CHM	Chemicals and chemical products
5	PTR	Coke, refined petroleum, and nuclear fuel
6	CSTR	Construction
7	EDU	Education
8	ELEQ	Electrical and optical equipment
9	ELCT	Electricity, gas, and water supply
10	FINC	Financial intermediation
11	FOOD	Food, beverages, and tobacco
12	HLTH	Health and social work
13	HTLS	Hotels and restaurants
14	LND	Inland transport
15	LTHR	Leather, leather, and footwear
16	MCHN	Machinery, Nec
17	MFG	Manufacturing, Nec; recycling
18	MNS	Mining and quarrying
19	OSRV	Other community, social and personal services
20	MRLS	Other non-metallic mineral
21	OTRS	Other supporting and auxiliary transport activities; activities of travel agencies
22	PST	Post and telecommunications
23	HHLD	Private households with employed persons
24	GVT	Public admin and defense; compulsory social security
25	PAP	Pulp, paper, printing, and publishing
26	ESTA	Real estate activities
27	LSNG	Renting of M&Eq and other business activities
28	RTL	Retail trade, except of motor vehicles and motorcycles; repair of household goods
29	PLST	Rubber and plastics
30	VHCS	Sale, maintenance, and repair of motor vehicles and motorcycles; retail sale of fuel
31	CLTH	Textiles and textile products
32	VHCL	Transport equipment
33	WTR	Water transport
34	TRD	Wholesale trade and commission trade, except of motor vehicles and motorcycles
35	WOOD	Wood and products of wood and cork

**Table 5:** Definition of symbols used in equations

Symbol	Definition	Dimension	Used in equation
Subscripts			
*i*	Country subscript	41 countries: see Table 3	
*s*	Sector subscript	35 sectors: see Table 4	
*t*	Time subscript	15 years: 1995–2009	
Vectors and matrices			
*T*	Territorial emissions		
*T*_*is*_	… in sector *s* of country *i*	1 × 1	(1)
*T*_*i*_	… in country *i*	1 × 1	(3)
*e*	Emission intensity of output		
*e*_*is*_	… in sector *s* of country *i*	1 × 1	
***e***_***i***_	… in all sectors of country *i*	35 × 1	(1)
*x*	Output		(3)
*x*_*is*_	… in sector *s* of country *i*	1 × 1	
***x***_***i***_	… in all sectors of country *i*	35 × 1	
*ε*	Emission intensity of value added		(1)
*ε*_*is*_	… in sector *s* of country *i*	1 × 1	(3)
*z*	Value added		
*z*_*is*_	… in sector *s* of country *i*	1 × 1	
*C*	Consumption-based emissions		(1), (5)*
*C*_*is*_	… in sector *s* of country *i*	1 × 1	
*C*_*i*_	… in country *i*	1 × 1	
*φ*	Emission intensity of final demand		(1)
*φ*_*is*_	… in sector *s* of country *i*	1 × 1	
$\overline{\varphi}$	… in average over all sectors and all countries	1 × 1	
***φ***_***i***_	… in all sectors of country *i*	35 × 1	(2)
${\overline{\boldsymbol{\varphi}}}_{\boldsymbol{s}}$	… in average per sector over all countries	35 × 1	(3)
*y*	Final demand		
*y*_*is*_	… in sector *s* of country *i*	1 × 1	
***y***_***i***_	… in all sectors of country *i*	35 × 1	(2), (6)*
*NCO*2*XP*	Net CO_2_ exports		(4), (9)*, (10)*, (11)*, (12)*
*NCO*2*XP*_*i*_	… from country *i*	1 × 1	(3), (4), (7)*, (8)*
***A***	Input-output coefficients		
***A***_***i***_	… in country *i*	35 × 35	(4), (7)*, (8)*, (9)*, (10)*, (11)*, (12)*
*XP*	Exports (of goods and services)		
***XP***_***i***_	… from all sectors of country *i*	35 × 1	
***XP***_***ij***_	… from all sectors of country *i* to country *j*	35 × 1	(2)
*MP*	Imports (of goods and services)		(3)
***MP***_***i***_	… in all sectors of country *i*	35 × 1	
Auxiliary matrices			
***I***	Identity matrix	35 × 35	(3)
***u***	Vector of ones	35 × 1	(4), (9), (10), (11), (12)

Note: Some elements that appear in the equations are time specific as indicated by a *t* subscript. These elements are not explicitly listed in this table, as they are equivalent to their counterpart without time dimension. * denotes the equations in which the elements are time specific

## Appendix 2. Robustness checks

**Table 6:** Effects on emissions per value added and output, controlling for sector structure, excluding smallest observations (unweighted)

Dependent variable	EIVA	EID	EI exports	EI exports
Equation number	(5)	(6)	(7)	(8)
ln(income)	− 0.854***	− 0.819***	− 0.733***	− 0.754***
	(0.046)	(0.020)	(0.027)	(0.014)
ln(income)^2^	0.002	0.013**	0.011	-
	(0.012)	(0.005)	(0.007)	
Rents coal	0.043**	0.038***	0.022**	0.033***
	(0.018)	(0.008)	(0.010)	(0.004)
Rents oil	0.018	0.016***	0.028***	0.033***
	(0.011)	(0.005)	(0.006)	(0.004)
Rents gas	− 0.007	− 0.019***	− 0.020***	− 0.020***
	(0.013)	(0.006)	(0.007)	(0.006)
Trade	− 0.261	− 0.078	− 0.210***	− 0.355***
	(0.168)	(0.073)	(0.075)	(0.043)
Kyoto	− 0.054	− 0.035**	− 0.031*	− 0.025*
	(0.034)	(0.015)	(0.019)	(0.013)
Country FE	YES	YES	YES	YES
Partner-year FE	NO	NO	NO	YES
Sector-year FE	YES	YES	NO	NO
Year FE	NO	NO	YES	NO
Weights	NO	NO	NO	NO
*N*	14,602	14,760	420	8070
*R*^2^	0.818	0.889	0.982	0.960
*R*^2^ within	0.033	0.132	0.719	0.299

Note: Standard errors in parentheses. */**/***: significant at 10/5/1%. All regressions are unweighted but the smallest 25% observations are removed

**Table 7:** Effects on sector structure, excluding smallest observations (unweighted)

Dependent variable	Exports (separate)	Imports (separate)	Bilateral Exports (joint)	Bilateral Imports (joint)	Bilateral Exports-Imports
Equation number	(9)	(10)	(11a)	(11b)	(12)
ln(income)	0.037**	0.050***	− 0.117***	− 0.034***	− 0.092***
	(0.015)	(0.011)	(0.009)	(0.009)	(0.012)
ln(income)^2^	− 0.005	− 0.008***	-	-	-
	(0.004)	(0.003)			
Rents coal	0.008	− 0.000	0.008**	− 0.004	0.013***
	(0.006)	(0.004)	(0.004)	(0.004)	(0.005)
Rents oil	0.003	− 0.005**	− 0.010***	0.001	− 0.009***
	(0.003)	(0.002)	(0.002)	(0.002)	(0.003)
Rents gas	0.003	0.004	− 0.002	− 0.003	− 0.001
	(0.004)	(0.003)	(0.003)	(0.003)	(0.004)
Trade	0.291***	− 0.041	0.303***	− 0.132***	0.409***
	(0.042)	(0.042)	(0.029)	(0.029)	(0.037)
Kyoto	0.011	0.021***	− 0.020***	− 0.003	− 0.017*
	(0.010)	(0.007)	(0.008)	(0.007)	(0.010)
Country FE	YES	YES	YES	YES	JOINT
Partner FE	NO	NO	YES	YES	JOINT
Year FE	YES	YES	YES	YES	NO
Weights	NO	NO	NO	NO	NO
*N*	420	420	16,815	16,815	8070
*R*^2^	0.936	0.913	0.629	0.629	0.347
*R*^2^ within	0.199	0.116	0.028	0.028	0.051

Note: Standard errors in parentheses. */**/***: significant at 10/5/1%. All regressions are unweighted, but the smallest 25% observations are removed

Tables 8 and 9 provide a robustness test for the results in 1,1. In Table 8, we replace the Kyoto index used in the main text by the CO_2_ index (Sauter 2014). However, we note that Sauter’s index is not available for major economies (USA, China, Brazil, and Indonesia; see the number of observations). Therefore, we repeat the estimations from Table 1 for the restricted country sample and report them in Table 9. We find that the change in the country sample affects the Kyoto coefficients significantly for Eqs. (5) to (7).

We proceed similarly to provide a robustness check for the results in Table 2. We repeat the estimations from Table 2 in Table 10 using Sauter’s index, and in Table 11 for the same restricted sample but with the Kyoto index.

**Table 8:** Effects on emissions per value added and output, controlling for sector structure, using CO_2_ index instead of Kyoto

Dependent variable	EIVA	EID	EI exports	EI exports
Equation number	(5)	(6)	(7)	(8)
ln(income)	− 0.727***	− 0.748***	− 0.736***	− 0.653***
	(0.052)	(0.026)	(0.026)	(0.012)
ln(income)^2^	− 0.013	0.000	0.026***	-
	(0.011)	(0.006)	(0.006)	
Rents coal	0.003	0.002	0.032**	0.015*
	(0.027)	(0.013)	(0.016)	(0.008)
Rents oil	0.007	0.003	0.016***	0.024***
	(0.011)	(0.005)	(0.005)	(0.005)
Rents gas	− 0.009	− 0.013**	− 0.010*	− 0.018***
	(0.012)	(0.006)	(0.006)	(0.005)
Trade	− 0.264	− 0.142	− 0.104	− 0.334***
	(0.183)	(0.091)	(0.070)	(0.032)
CO_2_ index	0.213**	− 0.024	− 0.020	0.099***
	(0.092)	(0.045)	(0.047)	(0.020)
Country FE	YES	YES	YES	YES
Partner-year FE	NO	NO	NO	YES
Sector-year FE	YES	YES	NO	NO
Year FE	NO	NO	YES	NO
Weights	YES	YES	YES	YES
*N*	17,420	17,851	525	8922
*R*^2^	0.852	0.889	0.985	0.972
*R*^2^ within	0.145	0.215	0.762	0.026

Note: Standard errors in parentheses. */**/***: significant at 10/5/1%. All regressions are weighted by VA, output, or exports

**Table 9:** Effects on emissions per value added and output, controlling for sector structure, using Kyoto but same sample as if CO_2_ index was used

Dependent variable	EIVA	EID	EI exports	EI exports
Equation number	(5)	(6)	(7)	(8)
ln(income)	− 0.701***	− 0.757***	− 0.743***	− 0.640***
	(0.050)	(0.025)	(0.025)	(0.012)
ln(income)^2^	− 0.009	0.002	0.028***	-
	(0.011)	(0.006)	(0.006)	
Rents coal	0.001	0.007	0.037**	0.011
	(0.027)	(0.013)	(0.016)	(0.008)
Rents oil	0.008	0.004	0.018***	0.021***
	(0.011)	(0.005)	(0.005)	(0.005)
Rents gas	− 0.010	− 0.013**	− 0.010*	− 0.018***
	(0.012)	(0.006)	(0.005)	(0.005)
Trade	− 0.268	− 0.143	− 0.099	− 0.328***
	(0.183)	(0.091)	(0.069)	(0.032)
Kyoto	− 0.091***	− 0.070***	− 0.063***	− 0.047***
	(0.032)	(0.016)	(0.016)	(0.010)
Country FE	YES	YES	YES	YES
Partner-year FE	NO	NO	NO	YES
Sector-year FE	YES	YES	NO	NO
Year FE	NO	NO	YES	NO
Weights	YES	YES	YES	YES
*N*	17,420	17,851	525	8922
*R*^2^	0.644	0.301	0.985	0.971
*R*^2^ within	0.008	0.001	0.024	0.022

Note: Standard errors in parentheses. */**/***: significant at 10/5/1%. All regressions are weighted by VA, output, or exports

**Table 10:** Effects on sector structure, using CO_2_ index instead of Kyoto

Dependent variable	Exports (separate)	Imports (separate)	Exports (joint)	Imports (joint)	Exports-Imports
Equation number	(9)	(10)	(11a)	(11b)	(12)
ln(income)	0.090***	0.037***	− 0.073***	0.061***	− 0.105***
	(0.016)	(0.013)	(0.013)	(0.013)	(0.010)
ln(income)^2^	− 0.001	− 0.005*	-	-	-
	(0.004)	(0.003)			
Rents coal	0.042***	0.013*	− 0.015*	0.001	− 0.025***
	(0.010)	(0.008)	(0.009)	(0.009)	(0.007)
Rents oil	− 0.004	− 0.005*	− 0.017***	0.013***	− 0.025***
	(0.003)	(0.003)	(0.003)	(0.003)	(0.003)
Rents gas	0.004	0.002	0.007**	− 0.012***	0.013***
	(0.003)	(0.003)	(0.003)	(0.003)	(0.003)
Trade	0.124***	0.068*	0.143***	− 0.176***	0.267***
	(0.042)	(0.036)	(0.036)	(0.036)	(0.027)
CO_2_ index	− 0.075***	− 0.038	0.047*	− 0.035	0.109***
	(0.029)	(0.023)	(0.025)	(0.025)	(0.020)
Country FE	YES	YES	YES	YES	JOINT
Partner FE	NO	NO	YES	YES	JOINT
Year FE	YES	YES	YES	YES	NO
Weights	YES	YES	YES	YES	YES
*N*	525	525	18,309	18,309	8864
*R*^2^	0.916	0.906	0.912	0.912	0.473
*R*^2^ within	0.251	0.067	0.088	0.088	0.119

Note: Standard errors in parentheses. */**/***: significant at 10/5/1%. All regressions are weighted by trade flows

**Table 11:** Effects on sector structure, using Kyoto but same sample as if CO_2_ index was used

Dependent variable	Exports (separate)	Imports (separate)	Exports (joint)	Imports (joint)	Exports-Imports
Equation number	(9)	(10)	(11a)	(11b)	(12)
ln(income)	0.081***	0.033***	− 0.068***	0.057***	− 0.093***
	(0.015)	(0.013)	(0.012)	(0.012)	(0.010)
ln(income)^2^	− 0.001	− 0.006**	-	-	-
	(0.004)	(0.003)			
Rents coal	0.045***	0.014*	− 0.016*	0.002	− 0.028***
	(0.010)	(0.008)	(0.009)	(0.009)	(0.007)
Rents oil	− 0.003	− 0.005*	− 0.017***	0.013***	− 0.026***
	(0.003)	(0.003)	(0.003)	(0.003)	(0.003)
Rents gas	0.005	0.003	0.006*	− 0.012***	0.012***
	(0.003)	(0.003)	(0.003)	(0.003)	(0.003)
Trade	0.124***	0.068*	0.140***	− 0.169***	0.255***
	(0.043)	(0.036)	(0.036)	(0.036)	(0.027)
Kyoto	0.008	0.019**	− 0.013	− 0.023**	0.000
	(0.010)	(0.008)	(0.010)	(0.010)	(0.008)
Country FE	YES	YES	YES	YES	JOINT
Partner FE	NO	NO	YES	YES	JOINT
Year FE	YES	YES	YES	YES	NO
Weights	YES	YES	YES	YES	YES
*N*	525	525	18,309	18,309	8864
*R*^2^	0.915	0.906	0.913	0.913	0.471
*R*^2^ within	0.002	0.002	0.002	0.002	0.001

Note: Standard errors in parentheses. */**/***: significant at 10/5/1%. All regressions are weighted by trade flows

## Footnotes

1 If a commodity is imported, repackaged, and exported, emissions are counted as if traded twice. In this sense, there is double counting and the share of traded emissions is overestimated.

2 Time subscripts are omitted to keep the notation as light as possible but will be added in the modelling equations of the “Determinants of emission intensities” section.

3 The calculation of emission data for water transport is challenging given the wide range of boat types (Sims et al. 2014). We rely on the data provided in the WIOD.

4 Note that sectors *s* have been stacked in (column) vectors for each country *i*. For example: ${\boldsymbol{x}}_{\boldsymbol{i}}=\left({x}_{i1}\kern0.5em \cdots \kern0.5em {x}_{is}\kern0.5em \cdots \kern0.5em {x}_{iS}\right)^{\prime }$. We consider all vectors as column vectors and add transpose symbols (′) where needed. We use bold font to denote vectors and matrices.

5 For a recent analysis of China’s carbon intensity, see Huang (2018).

6 Alternatively, we also implemented a two-stage strategy in which each endogenous variable (the fuel rents and trade) is instrumented by the corresponding interaction as defined in Eq. (13). In order to avoid cross-influences of the multiple instruments, the first-stage regressions are conducted by including only the corresponding interaction term. Linear predictions are then introduced in the second-stage equation and standard errors are thereafter adjusted by applying the correct mean squared error term. The results of this alternative (available on request) approach yield similar results to those displayed in the paper.

7 This result is in line with Karakaya et al. (2019), who identify an environmental Kuznets curve for production-based emissions, but no evidence in favor of a Kuznets curve for consumption-based emissions.
